# Measuring the intensity of rural livelihood diversification strategies, and Its impacts on rural households’ welfare: Evidence from South Gondar zone, Amahara Regional State, Ethiopia

**DOI:** 10.1016/j.mex.2023.102191

**Published:** 2023-04-18

**Authors:** Ferede Mengistie Alemu

**Affiliations:** Debre Tabor University, Department of Economics, Ethiopia

**Keywords:** Livelihood diversification, Households’, Welfare, South Gondar, Measuring the intensity of income diversification in rural dwellers by using *Simpsons Index of Diversity (SID)*

## Abstract

*In Ethiopia, rural households are under risk in the sustainability of sources of income and forced to involve in diversified livelihoods because of the uncertain nature of the agricultural sector. Therefore, the aim of the study is to measure the livelihood diversification strategies and its impacts on rural households’ welfare. The study used cross-sectional data, which is quantitative and qualitative in nature. Through the multistage sampling technique, the study selected 398 samples from the south Gondar zone. The data was analyzed through descriptive statistics, and Tobit and multiple linear regression models. The descriptive statistics analysis showed that income from crop and livestock productions are the most important contributors (97.74%) to livelihood diversification in the study area. The Simpsons Index of Diversity is (0.4) showing that there is a lower livelihood diversification in the study area. The Tobit model regression results showed that education, family size, irrigation, soil conservation, extension service, livestock, and infrastructure facility affect the intensity of livelihood diversification. Furthermore, the multiple regression result revealed that livelihood diversification has a positive impact on the welfare of rural households. The study concludes that to improve the livelihood and welfare of rural households, diversified livelihood strategies should be enhanced by facilitating farm activities through access to inputs, irrigation schemes, and infrastructure and off-farm activities.*

*This article applied the Simpsons Index of Diversity (SID) to estimate the intensity of income diversification.*

• *The method helps to measure the amount of diversified income and its effect on the welfare of rural dwellers.*
• *The study helps to identify the common livelihood strategies and their relevance to improving rural living style.*

## Graphical abstract

**Figure:**
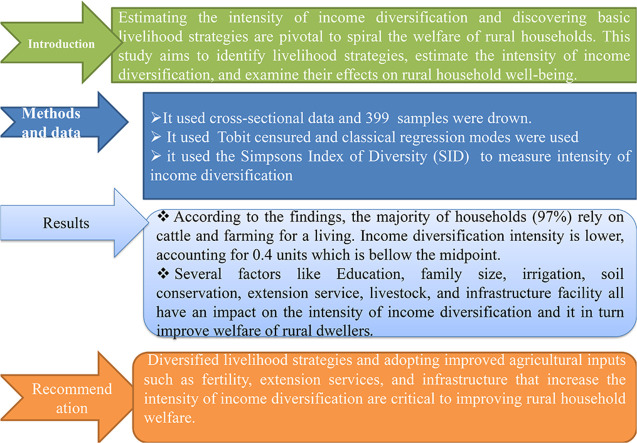

Specification table

**Table:** 

Subject area:	Economics welfare
Major specific subject area:	Estimating the intensity of income diversification, welfare and livelihood strategies
Method name:	Measuring the intensity of income diversification in rural dwellers by using *Simpsons Index of Diversity (SID)*
Name and reference of original method:	Gregorius HR, Gillet EM. Generalized Simpson-diversity. Ecological Modelling. 2008 Feb 24; 211(1–2):90–6.
Resource availability:	The data designed, collected and organized by the author. It is available from the author in excel forms. The data attached to the journal when requested by the publisher.

## Introduction

Agriculture is the principal source of capital for the investment of other sectors and reinvestment of itself in any developing country's economy. It contributes to the sustainable livelihood of most rural households in Sub-Saharan Africa  (SSA) [1]. It has a significant opportunity for sustaining growth, alleviating poverty, and improving food security [2]. In smallholder farming, agriculture dominates the largest share in Sub-Saharan African countries, which accounts for approximately 15% of GDP [3]. It remains important in driving economic transformation, sustainable livelihoods,  sustainable climate conditions and achieving development in developing countries  [4,5]. Agriculture in developing countries is the source of trade and foreign exchange, food consumption, a factor of production, and market contributions. However, it is unproductive and does not yield the expected outcome for the economy [6].

In Ethiopia, out of the total smallholders, 83% are engaged in farm strategies, while only 27% are engaged in non-farm/off-farm economic enterprises. Off-farm employment provides supplemental income, and allowing farmers to spend more on necessities such as food, education, clothing, and health care services [7,8]. Food security exists when all people have physical and economic access to enough nutritious food at all times to meet their dietary needs and food preferences for an active and healthy life [7]. However, Ethiopian agriculture is subsistence in nature; the land is fragmented, highly degraded, and rain-fed, making it incapable of accommodating the growing population pressure; for achieving food sustainability and reduce the uncertainties farmers are engaged in diversified livelihood strategies [9].

Livelihood diversification is comparatively better off smallholders primarily by exploiting opportunities and synergies between farm and off-farm activities  [10]. Income diversification is a collection of various livelihood strategies and social support capabilities used by households to improve their standard of living [7,[11], [12], [13], [14]]. In the rural African economy, we consider income diversification as the norm because it is essential to reduce the agriculture crisis drive from its uncertain nature  [15,16]. Agroforestry adoption and off-farm income generating activity involvement incidences jointly determined significantly and positively by the comparative economic return of adopting the cooperative activities  [17]. In Ethiopia, livelihood diversification strategies rely on farm, and off-farm activities especially, farm livelihood is the most commonly used livelihood strategies [18,19]. To realize rural households' well-being we must realize the expansion of livelihood diversification  [20]. Livelihood diversification is a strategy that can make better farmers’ income and endorse sustainable land management practices in Ethiopia [21]. According to empirical evidence, the farming sector employs roughly two-thirds of the country's total labor force and provides a living for roughly 90% of the rural population  [22]. To increase the income diversification of rural households, the Policymakers have centered on asset endowment during designing plans for agricultural and non-agricultural schemes [23]. The level of education, dependency ratio, access to irrigation, and household urban linkage significantly affected diversification of livelihoods  [24]. Livelihood diversification is significant to reduce rural poverty, and the empirical result showed that households that have diversified livelihoods were 9% better off than those that were not diversified in terms of poverty. Someone should strongly consider policies aimed at increasing the income generation ability of the household  [4]. Income diversification is directly correlated with shock related factors and it positively impacts welfare [25]. Livelihood diversification strengthens the rural society to be compelled with sorted off-farm and on-farm economic activities and it improves the wellbeing of society by increasing households' income source and reducing poverty [26].  A Wide range of income-generating activities improve the well-being of the rural poor through increasing the income source of poor households [4,27]. In most case welfare represented by consumption expenditure and it improved by Consumer and business sentiments [28].

Several studies failed to measure livelihood diversification and how it can improve the well-being of rural households at the micro-level. To sustain the improved well-being of rural households and plan an effective welfare maximization program, the study focused on identifying the common livelihood strategy, measuring livelihood diversification, and its impacts on the well-being of rural households. This is because the state of household livelihood strategies, measuring livelihood strategies, and the welfare effect of income diversification in the south Gondar zone have received little attention. Therefore, the study focused on: (1) investigating rural households' livelihood strategies, (2) estimating livelihood diversification and its determinants, and (3) investigating the effect of income diversification on rural households' welfare. Based on the above objectives, the study suggested policy implications aimed at improving the welfare of rural households and expanding rural livelihood diversified strategies.

## Literature review

The literature agrees that the driving forces of diversification operate at different levels. Responses of individuals and households to incentives for livelihood diversification categorized into two broad grouping: Various "push" and "pull" factors, as well as socioeconomic factors, influence a household's sustainable livelihood diversification  [16]. Livelihood diversification is crucial to improve the health status of households. A diversified livelihood had a profound influence on positive self-perceived physical health [29,30]. Rural households still predominantly pursue agricultural based livelihood strategies through agricultural intensification, and diversification [31]. Livelihood strategies are intentional in presenting sympathetic livelihood outcomes, including increased income, enhanced productivity, food security realization, poverty alleviation, and better conservation of natural resources [32,33]. Livelihood diversification is one mechanism of enhancing environmental sustainability but the adoption of diversified activities is strongly influenced by the age and education of the household head, the number of earning family members, social network, and government donation [34,35].

A good quality of life is synonymous with well-being. Furthermore, well-being is divided into two categories: material well-being and social well-being. Material well-being entails being strong, being in the right frame of mind, and looking good, whereas social well-being entails caring for and settling children, having self-respect, peace and good relations in the family and community, and having security, which includes civil peace, a safe and secure environment, personal and physical security, and confidence in the future [36,37]. The level of well-being is determined by household consumption expenditure and livelihood diversification [38,39]. The findings have important policy implications, implying that programs aimed at engaging people in other income-generating activities would supplement their income sources [4,20].

Livelihood diversification is an act of household members construct a diverse portfolio of activities and social support capabilities in their struggle for survival and in order to improve their standards of living [11]. It refers to individuals' and households' efforts to find new ways to increase income and reduce vulnerability to various livelihood shocks [32]. Livelihood diversification can occur through both agricultural diversification (the production of multiple crops or high-value crops) and non-agricultural livelihood diversification (the establishment of small businesses or the selection of non-agricultural sources of income such as casual labor or migration) [13,40].

Several factors affect livelihood diversification such as the education level of the household head, land size of the household, annual income of the household head, membership of households in the organization, credit utilization, and access to extension services were significant in determining the livelihood diversification of households in the study area. On the other hand, the age and family size of households were found to be negatively correlated to the household level of livelihood diversification [41]. Livelihood diversification has both short-run consumption gain and long-run wealth creation effect [38].

## Methodology

### Description of the study area

**Fig. 1:**
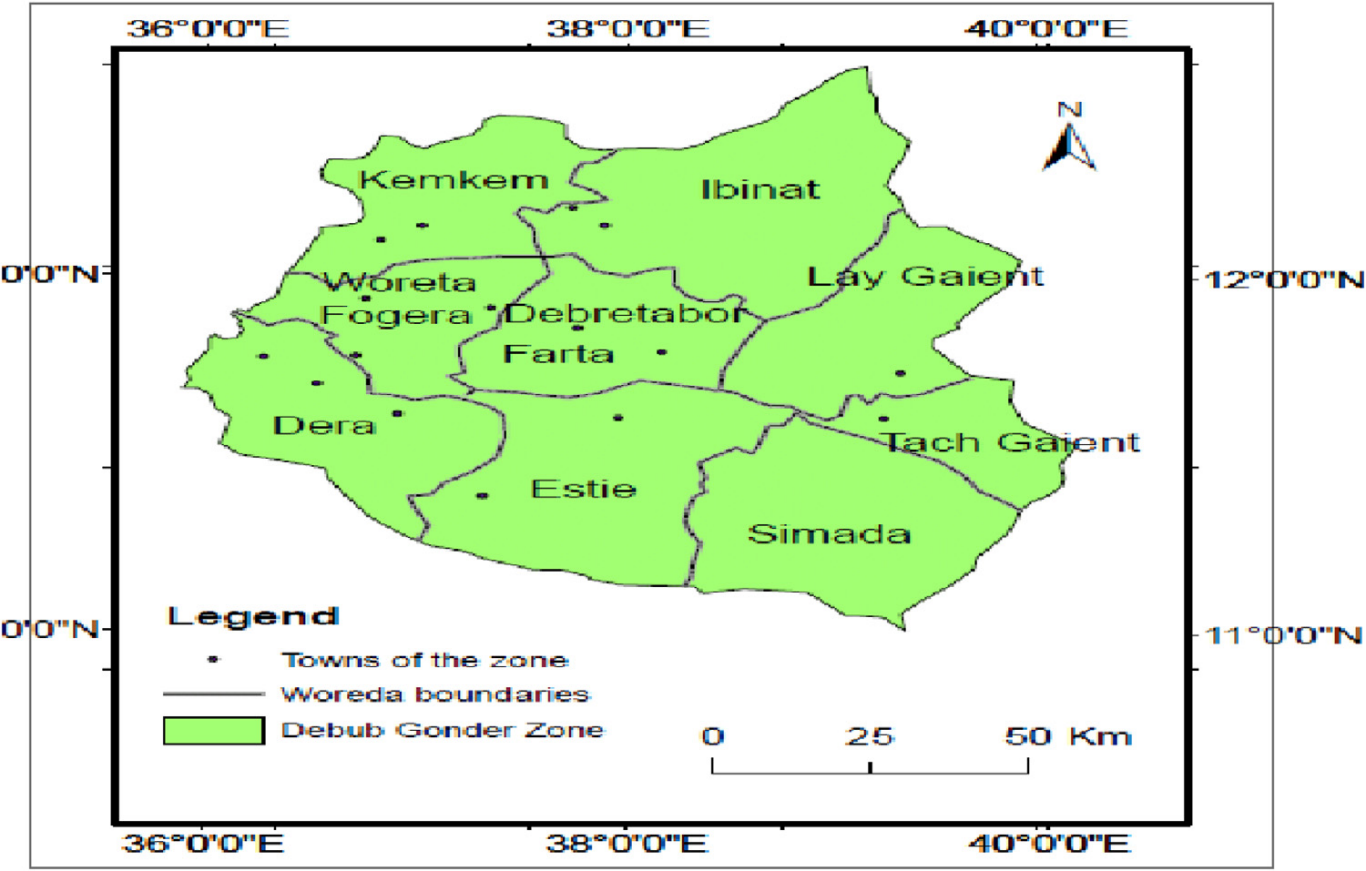
Map of the study area.

The study was conducted in the South Gonder Zone, **South Gondar**  (or **Debub Gondar**) is a Zone in the Ethiopian  Amhara Region. This zone is named for the city of Gondar, which was the capital of Ethiopia until the mid-19th century, and has often been used as a name for the local province. South Gondar is bordered on the south by East Gojjam, on the southwest by West Gojjam and Bahir Dar, on the west by Lake Tana, on the north by North Gondar, on the northeast by Wag Hemra, on the east by North Wollo, and on the southeast by South Wollo the Abbay River separates South Gondar from the two Gojjam Zones. (Fig. 1)

### Sampling techniques and sample size determination

To investigate the determinants of rural household livelihood diversification, measuring the livelihood diversification and its impact on household wellbeing in the south Gondar zone, multistage stratified simple random sampling technique was used to select samples from the targeted households. In the first stage, South Gondar was selected purposively. In the second stage, six Woredas were taken randomly from 13 Woredas of the Zone. In the third stage, 398 respondent households were selected from 13 Kebeles randomly. Simple random sampling is important to select samples from the population in such a way that every combination of samples is equally likely to be the sample selected [42]. A proportional approach is used to determine the sample size required from each targeted sample. Yamane's (1967) the simplified formula is used to determine the required sample size at a level of precision (e) of 5% is recommended in order to get a sample size that represents the true population.

$$n=\frac{N}{1+N{\left(e\right)}^{2}}$$

Where n is the representative sample size, N is the total number of households in south Gondar zone. The sample was determined as:-

$$n=\frac{70,785}{1+70,785*0.05^{2}}=397.752335463=398$$

### Model specification

There are different methods of estimating household livelihood diversification, such as the Simpsons Index of Diversity (SID, Herfindahl, Gibbs, and Martin's indexes, etc., but the Simpsons Index of Diversity (SID) is best to show the share of non-farm income in total household income.

In this study, the Simpsons Index of Diversity (SID) was used to estimate the level of income diversification among farm households in the south Gondar zone. The SID considers both the number of income sources and how evenly the distributions of income are made between the various sources  [43]. This justifies the use of the SID in this study over other diversification measures, such as Herfindahl, Shannon, and others. The Simpsons Index of Diversity (SID) was used in this study to estimate livelihood diversification among farm households in the south Gondar zone.

(1) $$\text{SID}=\;1-\sum_{i=1}^{n}p_{i}^{2}$$

In Eq. (1) where SID is Simpsons Index of Diversity, n is number of income sources, $p_{i\;}$ is Proportion of income coming from the$\;i^{th}$ income source. The value of Simpsons Index of Diversity (SID) ranges from Zero (0) to one (1). Thus, value zero (0) represents perfect specialization and value one (1) represents perfect livelihood diversification.

**In this study the SID model is expressed as in** (2)

(2) $$SID=1-\sum_{i=1}^{n}p_{i}^{2}$$

(3) $$p_{i}\;=1-\sum_{i=1}^{398}{\left(\frac{FCI}{THI}\right)}^{2}+\left({\left(\frac{CCI}{THI}\right)}^{2}+{\left(\frac{NRI}{THI}\right)}^{2}+{\left(\frac{LVI}{THI}\right)}^{2}+{\left(\frac{FWI}{THI}\right)}^{2}+{\left(\frac{NFI}{THI}\right)}^{2}+{\left(\frac{SEI}{THI}\right)}^{2}+\left(\frac{RI}{THI}\right){\;}^{2}\right)+{\left(\frac{OTHRI}{THI}\right)}^{2}$$

*From* (3*) where: FCI is food crops income, CCI is cash crops income, NRI is natural resource income LVI is Livestock income, FWI is farm wage income, NFI Non- is farm wage income, SEI is self-employment income, RI is remittance income, OTHRI is other income sources*. To determine the determinants of livelihood diversification of households in the case of south Gondar zone censured Tobit model was employed. The model can be specified as

$$\text{SID}=\left\{\begin{array}{c} SID*ifSID*\leq 0\\ SID*,\;ifSID*\geq 1 \end{array}\right\}\;\text{suppose}\;\text{it}\;\text{is}\;\text{interested}\;\text{in}\;\text{the}\;\text{expected}\;\text{value}\;E\left(SDI*\right)$$

The censured Tobit model was applied because of the censured value of the SDI, which is between zero and one. For such censured data, the Tobit model is too much effective for analysis. To effectively identify but it assumed that the model is (SDI*) then the presence of zeros in the dependent variable: Simpson's Index of Diversity for some respondents (thus showing no diversification) demands the use of the censored Tobit regression. The general formulation for Tobit specification is given in [44] write as in Eq. (4).

(4) $$y_{i}*=x_{i}\beta^{\prime }+e_{i}$$

Where, $y_{i}$ is dependent variable Simpson index of diversity, x_i  is explanatory variables,

$\beta^{\prime }$ is parameter, $e_{i}$ is error term.

$$\left\{\begin{array}{c} y_{i}*=0\;ify_{i}**\leq 0\\ y_{i}*,=y_{i}*ify_{i}*>0 \end{array}\right\}$$

$E\left(y_{i}/x_{i}\right)={x^{\prime }}_{i}B$ *This can be rewrite as*

$$E\left(y_{i}/{x^{\prime }}_{i}\right)=\frac{\gamma \left({x^{\prime }}_{i}\beta \right)}{\delta }{x^{\prime }}_{i}\beta +\delta \lambda_{i}$$

(5) $$\text{where},\;\lambda_{i}=\frac{\gamma \left[\left(0-{x^{\prime }}_{i}B\right)/\delta \right]}{1-\gamma \left[\left(0-\frac{{x^{\prime }}_{i}\beta }{\delta }\right)\right]}=\frac{\gamma \left(\frac{{x^{\prime }}_{i}\beta }{\delta }\right)}{1-\gamma \left(\frac{{x^{\prime }}_{i}\beta }{\delta }\right)}$$

The above Eq. (5) shows the marginal effect of censured Tobit regression model, where βisrepresents parameters and $x_{i}$ is represents explanatory variables.

On the other hand, the study was investigated the effects of livelihood diversification on households welfare. To examine the study, the welfare indicator is measured by the amount of consumption expenditure per adult equivalent [38]. Welfare index can be used to measure the level of well-being of households. Total household consumption expenditure per adult equivalent can calculate household welfare (wellbeing) in Eq. (6).

(6) $$\mathrm{THE}=\left(\frac{\mathrm{THE}}{n^{0.7}}\right)$$

Where, THE is total household expenditure, and “n” is household size, 0.7 is exponential formation representing other adults in a particular household [20]. Based on the welfare index of amount of consumption expenditure per adult equivalent the ordinary least square regression (OLS) model was used to show the impacts of livelihood diversification on welfare of rural households in the study area. This model showed the linear relationship between rural household income diversification and wellbeing. The OLS regression model can be specified as

WB=f(SID,othervariables)

(7) $$WB_{i}=\alpha +\beta x_{i}+\delta SDI_{i}+e_{i}$$

Where, $WB_{i}\;is\;i^{th}$ household welfare measured by consumption expenditure per adult equivalent, $x_{i}$ is other explanatory variables, $SDI_{i}$ is Simpsons Index of Diversity that shows income diversification of$\;the\;i^{th}$ household, and α, β, & δ are parameters and, $e_{i\;}$ is the error term.

## Result and discussion

### Descriptive analysis of socio-economic characteristics of sample households

Below is descriptive analysis of the socio-economic characteristics of sample households used in the study as presented in Table 1.

**Table 1:** The socioeconomic characteristics of sample Household Variable.

	Obs	Mean	Std. Dev.	Min	Max
SDI	398	0.4	.166	00.27	0.786
Age	398	49.99	15.23	18	110
Education	398	1.55	.725	1	4
Family size	398	5.912	2.04	1	11
Farm size	398	1.93	.782	0.25	4
Oxen	398	2.138	1.08	0	6
Distance to market	398	8.793	7.135	0	25
Livestock	398	11.611	11.203	0	85
Infrastructure	398	.382	.486	0	1
Price level	398	.947	.224	0	1

Source: Computed from own survey 2021.

The Simpsons Index of Diversity (SDI) measures the rate of livelihood diversification that ranges between 0 and 1. The value zero and one shows perfect specialization and livelihood diversification, respectively. In the study, the Average value of the Simpsons Index of Diversity (SDI) was 0.4, which is lower than the midpoint (Table 1). This showed that the average level of income diversification among rural households is exploited. Households in the study area mainly engage in crop production and livestock rearing and they are limited in off-farm activities. But their involvement in other on -farm activities such as forestry and gardens was limited because of inadequate cultivatable land and resource. They are also inactive to involve in off-farm income-generating opportunities like industries, and services in the study area. These incorrigible scenarios make households to inadequately involving in various portfolios of economic activities in the study.

Access to infrastructure contributes 0.382% to increase the household's opportunity to take part in diversified livelihood, whereas the stock of livestock and farm size contributes approximately 11% and 2%, respectively, for households to be involved in diversified livelihood. An increase in the price of the products has a 0.947% contribution to the participation of rural households in diversified livelihood strategies. It held this because of the extremely low prices of agricultural products, particularly onions, and tomatoes.

### Source of diversified livelihood strategies in the study area

The results in Table 2 showed which livelihood strategy is most commonly adopted by rural households to make safe their living standard. Among the set of diversified livelihood strategies, the rural households in the study area highly took part in crop production and livestock livelihood strategies, accounting for 97.7% of their income. In the study, 97.7% of rural households established their livelihood strategies only on the production of cropping and livestock. Only 2.3% of the rural households' income relied on other livelihood strategies such as mix of land rent and commerce, self-employment and mix of farming, cattle and gardening, which accounted for 1%, 0.50%, and 0.75%, respectively. The findings authenticate that farm and cattle livelihood strategies are decisive in escalating the income level of rural households.

**Table 2:** Livelihood strategies in the study area.

Livelihood strategies	Freq.	Percent	Cum.
Land rent and commerce	4	1.01	1.01
Crop production Farm & livestock	389	97.74	98.74
Crop, livestock, and garden	3	0.75	99.25
Self-employment	2	0.50	100.00
Total	398	100.00	

Source: Computed from own survey 2021.

### Determinants of livelihood diversification

The censured Tobit regression model has shown the relationship between income diversification and explanatory variables. The study used statistically significant variables for interpretation, discussion, conclusion, and recommendation.

The study focused on the determinants of livelihood diversification. Demographic factors such as age, education level, household size, marital status, and migration status are the basic determinants of livelihood diversification  [[18], [45], [46], [47], [48]]. In this study, the sex of the household head and family size positively affected the livelihoods diversification, whereas it negatively affected by the age of the household head. Male household heads have a high probability of participating in a diversified livelihood strategy than female household heads. Therefore, male household heads have involved in off-farm and farm activities than females because females allocate their time to non-productive economic activities. Educated households have enough knowledge, know how, experience and information about access to livelihood strategies and roles &challenges for participating in a diversified portfolio. As a result, education helps them to take part in diversified livelihood strategies.

The other variables that affected the livelihood diversifications are farm size, quality of land, and soil conservation [[16], [19], [49]] . In this study, farm size, land quality, and participation in soil conservation schemes positively affected the households’ livelihood diversification. Farm size plays a decisive implication to diversify the livelihood strategies of rural households by enlarging the access of rural households to allocate their land into intra-farm activities such as livestock, cropping, forestry, and gardening. Also, the quality of land and the active participation of rural households in soil conservation programs have often significant implications for expanding the diversification of livelihood strategies. Livelihood diversification strategies rely on the farm, and off-farm activities, but farm livelihood activities were the most common and heavily reliant on soil conservation. The income diversification rate of households with more fertile land is 0.089 units higher than households with less fertile land. Because when the land is overly fertile, households' ability to take part in various agricultural activities since it enforces them to use the land for a diversified cropping portfolio.

The critical factors in determining livelihood diversification are assets measured as livestock and crop production [[23], [43], [50]] . The result reviled that livestock is one stock of assets for rural households that are used as the base for one source of income-generating livelihood strategy. Livestock income is comprised of livestock sales, livestock products, and services. The total cost of livestock production was calculated by aggregating annual expenditures on cost items such as purchased fodder or straw, veterinary services, and hired labor. After deducting, the total annual cost from the gross livestock income is the net livestock income. The study showed extension services positively predisposed that the livelihood diversification. Access to enough extension services endows them with different information, knowledge, and skill about confrontation and prospects of diversified livelihood strategies. It results in changing the attitude of rural households to being risk lovers and participates in a diversified livelihood strategy.

Livelihood diversification has long been viewed as a risk-mitigation strategy in developing countries during the occurrence of weather and market fluctuations [7]. Off-farm agricultural activities play an important insinuation in stimulating economic growth. Human capital and infrastructure-related variables persuade households to take part in off-farm economic activities that in turn augments the households income [[18], [33], [35], [51]]. The result reviled that, stamping out entry barriers to off-farm activities (access to finance, markets, education, and infrastructure) and spreading out off-farm opportunities (micro and small enterprises) are playing a catalytic role to trim down rural poverty, and expanding the livelihood strategies. The direct and significant contribution of improved infrastructure, which comprises roads, electricity, schools, and hospitals, has played a significant role in increasing income diversification through increasing the different occupation accesses for households. Expansion of infrastructure (roads, markets, and credit access) boosts the employment opportunity to engage in self-employed, construction worker, security, and hotel service. An increase in the level of infrastructure in the study area leads to the expansion of various economic activities that are considered as the primary source of income generation, and it allows the rural poor society to achieve their well-being.

Another variable is the shock (natural incidence) that comprises flooding, illness, and acid rain during the household's productive season. It is statistically significant at the 1% level of significance and it has a negative impact on income diversification. The result in Table 3 showed that the level of income diversification of households with shock is 0.059 units less than the rates of income diversification existing in the condition without shock. It is a serious cause for peasants with subsistence agriculture, market and finance failure, and risk and uncertainty in the production of crops and livestock. Households experiencing with health issues or crop failure have a negative relationship with income diversification strategies.

**Table 3:** Determinants of livelihood diversification.

SDI	Coef.	St.Err.	t-value	*p*-value
Age of households	−0.001***	.001	−2.66	.008
Sex of households	.074***	.021	3.52	0.002
Education level	.02*	.01	1.89	.06
Family size	.001	.004	0.32	.749
Farm size	.031***	.011	2.82	.005
Number of Oxen	.001	.007	0.17	.868
Land Quality	.089**	.039	2.30	.022
Irrigation schemes	.021	.017	1.24	.216
Soil conservation	.077***	.02	3.92	0.001
Extension service	.043**	.018	2.42	.016
Credit access	.002	.017	0.12	.902
Soil Fertility	0.003	0.00	0.36	.719
Distance to market	−0.002**	.001	−2.19	.029
Livestock	.001*	.001	1.80	.073
Shock	−0.059***	.017	−3.44	.001
Infrastructure	.061***	.016	3.94	0.00
Price level	.023	.035	0.66	.51
Constant	.261	.06	4.32	0.00
var(e)	.022	.002		
Pseudo R^2^ = 67.32	Obs = 398			
Chi-square =96.34	Prob>chi^2^= 0.00			

Source: computed from own survey 2021.

“Notes: Standard errors in parentheses*** *p*<0.01, ** *p*<0.05, * *p*<0.1 indicts the level of significances of variables at different level of significance such as 1% 5%and 10%, respectively”.

### The effect of livelihood diversification on rural household's welfare

In the bellow Table 4, the regression result showed the direct relationship between household well-being and income diversification. Welfare is significantly affected by age of household head, family size, land fertility, irrigation schemes, and distance to the market and livelihood diversification. The study postulated that age of household head has an affirmative effect on households’ wellbeing. Aged household head has larger opportunity to get income from remittance, interest and others than the young households. The rational human beings are save more but consume less at youth age and consume more but save less at old age, therefore as households are becoming elder they consume much share of their assets which drive from wealth and early age saving. Precipitated income and consumption are negative functions of family size [52]. Encourage the use of irrigation schemes improved rural households' income and asset position by decrease liquidity constraint and addressee sustainable opportunities [53,54]. The study reviled that the Irrigation scheme plays constructive impacts in enhancing the welfare of households in the study area. Because irrigated households have higher incomes yielding and better welfare (per capita household consumption) than non-irrigated households. According to respondents' responses, irrigation enhanced households’ welfare apart from covering costs incurred for agriculture inputs, schools feed, shelter, and food. Why this apprehended is because irrigation augments households’ income obtained from cash, stable, and vegetable commodities production. But family size is depressingly affected households' well-being while it dwindle the total proceeds allocated for consumption per household. Large family size enhanced the welfare of households when there are different opportunities for households to participate in diversified livelihood strategies but the fact does not exist in the study area because of the low level of livelihood diversification. Also, in this study, land fertility has a positive contribution to enhancing the welfare of households by increasing the total output of farmland driven by the productive capacity of fertile lands. The marginal productivity of fertile land is exhibiting an increasing return and it contributes to accusation wealth in the long run and enhanced consumption in the short run.

**Table 4:** Effect of income diversification and other factors on household's welfare.

THE	Coef.	St.Err.	t-value	*p*-value
Age of household	144.914**	59.617	2.43	.016
Sex of household	273.444	2065.799	0.13	.895
Education level	434.771	1151.558	0.38	.706
Family size	−746.327*	423.403	−1.76	.079
Farm size	593.141	1164.378	0.51	.611
Oxen	−540.547	759.051	−0.71	.477
Irrigation	5619.052***	1971.089	2.85	.005
Extension service	2625.244	1839.45	1.43	.154
Credit Access	1243.633	1933.37	0.64	.52
Soil Fertility	7.418*	4.433	1.67	.095
Distance to market	−192.143*	115.533	−1.66	.097
Livestock	−11.009	53.869	−0.20	.838
Shock	1547.922	1890.315	0.82	.413
Price level	95.827	3622.76	0.03	.979
SDI	9722.044*	5125.792	1.90	.059
Constant	276.366	6302.325	0.04	.965
R^2^	0.93			
F-test	2.603			
prob>*F*	0.001			

Source: Computed from own 2021 survey.

“Notes: Standard errors in parentheses*** *p*<0.01, ** *p*<0.05, * *p*<0.1 indicts the level of significances of variables at different level of significance such as 1% 5%and 10%, respectively”.

Livelihood diversification has both short-run consumption gain and long-run wealth creation effect [38]. Participating in a wide range of income-generating activities to improve the well-being of the rural poor [4,27].

From Table 4, study directed that livelihood diversification has a considerable and encouraging impact on the welfare of rural households. Livelihood diversification is an act of households’ involvement in a wide range of economic commotion. Peasants are underprivileged and irritated to dedicate themselves to specific economic activity because of uncertain ventures and too much nature reliant on agriculture. Relying on this; rural households’ livelihood diversification improved their welfare. Because livelihood diversification minimized the failure of agriculture arises in natural and human-made risks and uncertainties. An increase in the participation of deprived rural households in diversified livelihood strategies has long been authenticated to improve the welfare of households. Developing livelihoods only solitary livelihood strategies, specifically, rain-fed cropping and livestock, have low contributions to enhancing the income and welfare of households than those whose livelihood relies on diversified livelihood strategies.

## Conclusion and recommendation

Agriculture is the major spring of livelihood strategy to enhance welfare of deprived societies in developing countries, including Ethiopia. Despite its critical role, the Ethiopian agriculture is subsistence in nature; it cannot absorb the dramatic population growth anxiety by agglomerating rural households’ livelihood diversification strategies. The result showed that crop and livestock productions have the leading contributions which account for (97.74%) of livelihood diversification strategies of rural households, while the remaining share of diversified livelihood contributed from other sources. According to the Simpsons Index of Diversity, the value is between zero and one: when the value is close to 1; it shows perfect diversification, and close to zero shows perfect specialization. In the study, the Simpsons Index of Diversity result (0.4) showed that there is low degree of livelihood diversification in the study area.

The tobit regression result revealed education, family size, irrigation, soil conservation, extension service, livestock, and infrastructure facility are positively influences the livelihood diversification. Also, the multiple regression result showed that livelihood diversification plays a synergetic role in improving the welfare of households in the short run. It can improvise welfare through decreasing the cost of risk and uncertainty by adopting various technological innovations. Therefore, the study concludes that welfare is positively affected by livelihood diversification strategy. The study recommends that to improve the welfare of rural dwellers, the government and the concerned body should intensifying and rehabilitate the off-farm livelihood diversification strategies by expanding irrigation, extension service, and technologies and installing off-farm livelihood diversification strategies through create integration between peasants and agriculture institutions, perhaps to establish agriculture product processing industry, in the study area.

## Declaration of Computing Interest

The authors declare that have no known competing financial interests or personal Relationships that could have appeared to influence the work reported in this paper.

## Data Availability

Data will be made available on request.
